# Expert Testimony1Read before Buffalo Medical Club, October, 1897.

**Published:** 1897-12

**Authors:** James W. Putnam

**Affiliations:** Buffalo, N. Y., Professor of nervous diseases University of Buffalo


### EXPERT TESTIMONY.[1]

JAMES W. PUTNAM, M. D., Buffalo, N. Y.,
Professor of nervous diseases University of Buffalo.

THE complexity of our civilisation has resulted in a minute division of labor and of necessity of special departments of knowledge. As a natural consequence, it has been found that in our courts questions continually arose which required the opinion of one specially skilled or trained in a particular branch of industry or science. And it has become customary to call upon men to give information to the court and jury which will lead to the interpretation of sworn facts from lay testimony. This is necessary and reasonable. It is an expedient to which all or any of us would resort, rather than rely upon our own judgment. For instance, if we were inclined to assist some one by giving him money for a diamond we would probably take the stone to an expert, or perhaps to two, for the purpose of having his expert opinion on the definite fact. Here is a crystal; the layman says it is a diamond. The

1. Read before Buffalo Medical Club, October, 1897.

testimony here asked for is expert. So is the testimony of the person skilled in the detection of counterfeit money, and so through the list of skilled arts.

Most important, however, is the expert medical witness. To him are submitted facts, from which he is asked for an opinion in a great variety of cases. Prominent are questions of legitimacy, divorce and rape ; questions of insanity, as affecting testamentary capacity and as a defense for crime ; questions concerning various obscure injuries ; questions which arise in life insurance cases— all of these questions of vital importance to the community are submitted to the medical profession for an expert opinion on the facts presented. Civilised society believes these questions cannot possibly be settled without our aid.

The honor paid to the physician is a great one when he is asked for an opinion on facts which involve life and property and justice. It is a curious fact that the method of obtaining this testimony has been so unsatisfactory as to render the professional expert appear more often in the psychic partisan rôle of professional advocate instead of a scientific judge.

Of late years there has been a growing distrust of all experts and a feeling against the admission of their testimony. To better show how authors view the subject I quote. Wharton on evidence, section 454, says :

When expert testimony was first introduced it was regarded with great respect. An expert was viewed as the representative of a science of which he was a professor, giving impartially his conclusions. Two conditions have combined to produce a material change in this relation.

1. In matters psychological, there is no hypothesis so monstrous that an expert cannot be found to swear to it on the stand and to defend it with vehemence.

2. Then the retaining of experts by a fee proportioned to the importance of their testimony is now as customary as is the retaining of lawyers.

Hence it is that, apart from the partisan character of their opinions, their utterances, now that they have as a class become the retained agents of the parties, have lost all judicial authority and are entitled only to the weight which sound and consistent criticism will award to the testimony itself. Lord Kenyon used the following language :

"Skilled witnesses come with such a bias on their minds to support the cause in which they are embarked that hardly any weight should be given to their evidence.

But that is an English opinion of English experts ; you may think we stand better over here. Let me disabuse you by quoting Judge Davis of the Supreme Court of Maine in the Neil case :

If there is any kind of testimony that is not only of no value, but even worse than that, it is, in my judgment, that of medical experts. They may be able to state the diagnosis of a case more learnedly, but upon the questions whether it had at a given time reached a stage that the subject of it was incapable of making a contract or irresponsible for his acts, the opinions of his neighbors of men of good common sense would be worth more than that of all the experts in the country.

Stephen, in his criminal law, p. 209, says : "I object to the proposition of referring scientific questions to them (experts)."

In this way has the medical profession of England and America dishonored the trust put upon them. Our judges say our opinions are bought and sold like any other commodity, and after it is given it is worth nothing.

What has brought about this discredit ?

Is it that we are less fair-minded and judicial, or is it that we are less honest than are the experts of France and Germany ? For in those two countries we find no such distrust.

A study of this question as we know it in Erie county may be interesting. A murder has been committed and the criminal has been apprehended. He cannot prove an alibi nor self-defense. The public know that the defense will be insanity and they know that physicians will be found who will swear they are experts in insanity and that the criminal is insane, and that other physicians will be employed by the district attorney who will swear that they are experts and that the man is sane and responsible. The system that makes this possible is not of the making of the physician. We can plead not guilty to the cause of this farce—we are the natural product of the system. In the first place, what qualifications are required by the court before the testimony of a given man is received as an expert in a given case ? Absolutely none. I have seen men qualify as experts who testified that they had graduated from some medical college a few years ago and had practised their profession ever since. It was not necessary for the witness to prove to the satisfaction of the court that he had more than ordinary knowledge of a special branch of medicine, that he had special claim to special knowledge which would entitle him to be believed rather than any other physician. On the contrary, everything with an M. D. attached passes as an expert, and is referred to by the eulogistic attorney, who would not employ him as a physician, as an eminent and distinguished member of his profession. I have seen the same physician an expert in questions of surgery and insanity, eminent in both. I have been in cases during trial and suddenly the attorney would say, "I want another expert." A messenger goes to the telephone, calls up an eminent gentleman, and tells him he is wanted as an expert in a case. The eminent gentleman comes into court, asks the lawyer what the case is and, strange to say, his opinion was just what the lawyer wanted. I do not say the expert was not honest, but that it was not surprising that his testimony was valueless. While there is nothing to prevent a man from rating himself an expert—so long as no qualification is required by the court—so long we will find men who will swear to anything.

What can we ourselves do individually to remedy this evil and restore the testimony of physicians to something of its former dignity?

It seems to me that the lawyer must be made to understand that when he approaches the doctor with a case, that he comes first to present the facts to him and then get his opinion and pay him for it. If that opinion is one that he can use, then employ him as a witness. If it is an opinion that he cannot use because it is adverse to him, let him, if the physician is a man whose services are required, be retained for advice and be paid without going on the stand.

This is not Utopian—this is practical, and just as profitable to the doctor in the long run as the other policy of taking whichever side is first presented. If we go on the witness stand as the result of careful study of a case, and have come to conclusions as a result of such study, our opinions are matured and we shall be fully able to maintain them.

In an experience with a large number of damage suits against railroads, I have adopted the following policy: I examine the case, report upon the facts as I find them and state what my opinion is. If the case comes to trial I usually find the physician on the other side is willing to admit my facts, but differs in his conclusions. I usually find in studying the cases that we are both somewhat influenced by our training and reading, and that it is impossible to agree. Does that prove that we are not honest or that the circumstances of our employment makes it impossible to see the case correctly? I believe that in the vast majority of cases we honestly differ, and that each of us is a healthy check upon the other.

In some cases I have come across most palpable frauds and have found most unscrupulous physicians who appeared as experts. They had to swear and swear hard, for their fee depended upon success. In many cases where expert testimony is given I have observed a certain shrewdness of physicians. They attempt to give evasive answers—to give half a truth, not the whole truth. They show they are partisans by their testimony. Their appearance on the stand is exceedingly entertaining, but it is not convincing. Some of the best exhibitions of mental gymnastics by experts have been proved to have very little weight with juries. The appearance of candor and fairness, a willingness to state or at least admit scientific truths which do not strengthen the side which is paying you, gives the witness greater authority with the jury when he gives his final opinion.

Let one consider the way in which an opinion is obtained. Each side prepares a wonderful question—the hypothetical question. Each attorney includes all the facts favorable to his side and suppresses all the unfavorable facts he dares. These questions vary in length from a document that takes ten minutes to read to one that will take two to three hours. The witness who has already made up his mind is asked for his opinion. The question is usually so adroitly framed that but one answer is possible, and to one question he answers sane, and to the question of the opposing side he must answer insane.

This was true in the Ingalls will case. In this case a question that took ninety minutes to read was propounded by the contestants. In this question certain sworn statements were grouped in such a manner that the answer insane was inevitable. The defendants of the will assumed another set of facts, which, if true, compelled me to state that the man was sane. My opinion as to whether Ingalls was sane or insane was never asked and never given. A physician could come at an opinion much more nearly correct if he were to have all the testimony placed before him given him to study and then asked for his opinion on the case.

Let him state in answer to a question that he has studied all the testimony and arrived at such and such an opinion based on such and such facts. Let him state the theory of insanity which is consistent with this judgment and let his opinion be tested in accordance with his theory by a rigid cross-examination. Such a method would do away with the hypothetical question which never truly states a case and would give the witness an opportunity to state his real opinion. By this method I do not believe that the evil of the present method would be removed. We would still have partisans—but the partisans would not be able to crawl behind the hypothetical question and say "I never said he was insane; I said the hypothetical question described an insane person. If I had been asked what I really thought I should have answered the other way."

One thing more. It has frequently been charged that experts will always swear as they are expected or employed to swear. This is a great exaggeration. It is my experience that in civil cases for damages that men's consciences are a trifle more elastic than common, and that they take wonderfully gloomy views of the consequences of a slight injury; but in criminal cases I believe that it is rare to find physicians willing to testify for the people, unless they are firmly convinced of the soundness of their opinion. It is a very mean man who will take the stand and swear a poor devil is sane and therefore guilty of murder if he is doubtful about it. I think the mental attitude of the expert for the defense is a trifle different. He feels that he is helping the under dog and doing nobody any harm if he is mistaken. As a general rule I think the experts for the people are more apt to be right than those for the defense.

I have two notable exceptions to this statement in mind at present—Guiteau and Prendergast. I believe both these men were insane, yet I think the experts believed that their execution was politically necessary. Whether they were right or wrong in this it is not my province to discuss.

That experts have acted as judges of testimony and have refused to appear for the people has occurred twice in my own experience, once in this county, when Ellen Hughes was tried for infanticide. The defense was insanity. The experts summoned by the district attorney believed she was insane and were not called to the stand.

In Wayne county, in the case of the People against Childs, the murderer of Nichols, Dr. MacDonald, Dr. Ford and myself were employed by the district attorney to look after the interest of the people. The testimony of lay witnesses was so weak in laying the foundation for insanity that we were sure of a victory for the people. We were not allowed to examine the prisoner till the third day, when we were all convinced that we were on the wrong side. We so reported to the district attorney and the judge. The trial was stopped then and there, and the prisoner was sent to Matteawan. Did that raise the standard of expert testimony in that county? Not a bit of it. Sold out, was what was whispered.

From my experience in the past seven years in criminal trials I want to emphasise that I think the cases are rare in which experts for the prosecution are not thoroughly in sympathy with their side. I think that it often occurs that witnesses will honestly differ, and I see no more reason for doubting their honesty of opinion on a given set of facts than for doubting the honesty of judges of the Court of Appeals. They do not always agree.

Finally, of what value is the testimony? According to the judges it is of very little; according to many jurymen it is of little. Nevertheless, it is necessary, and it is for us to give it or withhold it, so that when we do appear it shall be a guarantee that that side has our honest endorsement.

527 DELAWARE AVENUE.

